# Editorial: New Insights Into the Molecular Mechanism of Amyotrophic Lateral Sclerosis Pathogenesis

**DOI:** 10.3389/fnins.2022.953261

**Published:** 2022-06-24

**Authors:** Cheng Li, Renshi Xu

**Affiliations:** Department of Neurology, Jiangxi Provincial People's Hospital, First Affiliated Hospital of Nanchang Medical College, Clinical College of Nanchang Medical College, Nanchang, China

**Keywords:** molecular mechanism, genomics, pathogenesis, amyotrophic lateral sclerosis, proteomics

Amyotrophic lateral sclerosis (ALS) is a devastating neurodegenerative disease that is characterized by the degeneration of both upper motor neurons and lower motor neurons, leading to motor and extra-motor symptoms. Although ALS has a low prevalence, its high mortality makes it one of the most intractable diseases. Mean survival time from the onset is 3–5 years, but some people could live for more than 10 years while slowly losing mobility. The pathogenesis of ALS remains unknown; however, recent research suggests that genetic factors may play an important role.

The objective of the Research Topic “New Insights into the Molecular Mechanism of Amyotrophic Lateral Sclerosis Pathogenesis” was to gather original research articles and reviews illustrating the recent advances concerning the molecular mechanism of ALS pathogenesis. This Research Topic consists of four original articles.

Zhang et al. proposed 27 candidate loci that were most possibly linked with sALS, which were identified for further analysis employing sequenom massARRAY technology and DNA sequencing in a separate case/control cohort of 239 sALS patients and 261 control subjects of Han ancestry from mainland China (HACM) ethnicity. They discovered that the polymorphism rs2619566 located within the contactin-4 (CNTN4) gene, rs10260404 in the dipeptidyl-peptidase 6 (DPP6) gene, and rs79609816 in the inositol polyphosphate-5-phosphatase B (INPP5B) gene were strongly associated with sALS in subjects of Han ancestry of China mainland. They also demonstrated the polymorphisms of rs2619566, rs10260404, and rs79609816 may affect the splicing, transcription, and translation of CNTN4, DPP6, and INPP5B genes and may play roles in the pathogenesis of ALS.

Barton et al. used human pathology material from sporadic ALS patients, genetic ALS patients (carrying the C9orf72 mutation), and age- and sex-matched non-neurological controls to explore myelination at an RNA, protein, and structural level. They used electron microscopy to achieve (i) quantitative spatial profiling of the myelin basic protein (MBP) mRNA transcript, (ii) quantification of MBP protein, and (iii) the first quantitative structural assessment of myelination in ALS post-mortem tissues. Although significant dysregulation of subcellular transport of MBP mRNA was found in ALS patients compared with controls, there were no differences in MBP protein levels or myelination ultrastructure. They confirm that whilst there is a cell-autonomous mRNA transport defect affecting oligodendrocytes in ALS, it has no effect on myelin structure.

Xu et al. compared the transcriptomic profile of the anterior horns of the lumbar spinal cord (AHLSC) between SOD1G93A mice and their wild-type (WT) littermates. When comparing pre-symptomatic/symptomatic ALS mice to WT mice, they discovered that bone marrow stromal cell antigen 2 (BST2) was considerably greater in the AHLSC of pre-symptomatic/symptomatic ALS mice. Immunofluorescent staining further confirmed that BST2 is mainly expressed on microglia in the AHLSC of ALS mice. These findings support the view that immune-related neuroinflammation plays a role in the early stages of ALS pathogenesis, and that BST2 could be used to treat microglia-mediated neuroinflammation later in the disease.

Bigelow et al. explored the β-sitosterol β-D-glucoside (BSSG) model with a focus on motor function, and associated immunohistochemical markers. They used BSSG modeling to detect behavioral changes and immunohistochemical markers in mice. The results revealed that no changes in behavior were observed at any time point. Animals were processed for immunohistochemistry markers of substantia nigra integrity after behavioral testing. In the substantia nigra, immunohistochemistry indicated no alterations in the microglial marker Iba1 or the dopaminergic integrity marker tyrosine hydroxylase (TH) at any assessment point. The lack of any behavioral or immunohistochemical changes across groups implies an inability to duplicate earlier findings. Before the BSSG model can be used in preclinical studies, more research into the sources of variability in the model is required.

In summary, the articles presented in the Research Topic “New Insights into the Molecular Mechanism of Amyotrophic Lateral Sclerosis Pathogenesis” cover four different aspects of ALS research, expanding our understanding of the disease and provide a valuable source of information concerning the molecular mechanism of ALS pathogenesis.

## Author Contributions

All authors listed have made a substantial, direct, and intellectual contribution to the work and approved it for publication.

## Funding

This study was supported by grants the Committee of National Natural Science Foundation of China (30560042, 81160161, 81360198 and 82160255), Education Department of Jiangxi Province (GJJ13198 and GJJ170021), Jiangxi provincial department of science and technology ([2014]-47, 20142BBG70062, 20171BAB215022 and 20192BAB205043) and Health and Family Planning Commission of Jiangxi province (20181019) (All to RX).

## Conflict of Interest

The authors declare that the research was conducted in the absence of any commercial or financial relationships that could be construed as a potential conflict of interest.

## Publisher's Note

All claims expressed in this article are solely those of the authors and do not necessarily represent those of their affiliated organizations, or those of the publisher, the editors and the reviewers. Any product that may be evaluated in this article, or claim that may be made by its manufacturer, is not guaranteed or endorsed by the publisher.

